# Death from Chloroform, in Toronto, C. W.

**Published:** 1867-03

**Authors:** 


DEATH FROM CHLOROFORM, IN TORONTO, C. W.—We have received an account of a coroner's inquest upon the body of one John Gould, who died suddenly from inhalation of chloroform in the Toronto General Hospital, where he had been admitted for operation. It does not appear from the evidence that the administration was in any way in fault. The medical students appear to have been greatly excited and to have kicked in a window when it became evident that the patient was in danger. But small quantity of chloroform was given, and symptoms of danger were manifest very early after commencing inhalation. The jury returned a verdict, "that no blame can be attached to any of the officers of the hospital, but that the demonstrations by the students, and the crowded theatre, must have had a great influence upon the mind of the patient."

